# BDD/PPy Composites with Low Interfacial Resistance for Energy Storage and Theoretical Feasibility for Pollutant Sensing

**DOI:** 10.3390/nano16120755

**Published:** 2026-06-16

**Authors:** Shuhan Wang, Yifan Ren, Qinghai Yu, Jiarui Yang, Jiali Lin, Lingpei Shi, Yuanyuan Li

**Affiliations:** 1 School of Materials Science and Technology, China University of Geosciences (Beijing), Beijing 100083, China; shuhan_wang@email.cugb.edu.cn (S.W.); yifan_ren@email.cugb.edu.cn (Y.R.); jiarui_yang@email.cugb.edu.cn (J.Y.); jiali_lin@email.cugb.edu.cn (J.L.); lingpeishi@email.cugb.edu.cn (L.S.); 2464770458@qq.com (Y.L.); 2 School of Gemmology, China University of Geosciences (Beijing), Beijing 100083, China

**Keywords:** boron-doped diamond, polypyrrole, charge transfer resistance, theoretical sensing feasibility, bifunctional electrode

## Abstract

Self-powered integrated electrochemical systems require electrode materials that can simultaneously provide energy storage and sensing functions. Boron-doped diamond (BDD) electrodes have good chemical stability and a wide potential window, but their small specific surface area and slow interfacial charge transfer limit their use in such bifunctional applications. In this work, we prepared a three-dimensional porous BDD scaffold on titanium foam by hot-filament chemical vapor deposition, and then grew polypyrrole (PPy) layers on the scaffold by in situ oxidative polymerization. The polymerization time was varied from 8 to 20 h. The BDD/PPy composite obtained after 12 h showed an areal capacitance of 398.6 ± 15.2 mF/cm^2^ at 1 mA/cm^2^, which is about 5.8 times that of the porous BDD alone (67.9 mF/cm^2^). Its charge transfer resistance (*R*_ct_) was as low as 1.3 ± 0.1 Ω, among the lowest reported for BDD-based electrodes. The porous BDD framework provides ion diffusion pathways, while the PPy layer introduces pseudocapacitance. X-ray photoelectron spectroscopy reveals that the PPy layer contains pyrrolic –NH– groups, which are known to chelate various water pollutants (e.g., heavy metal ions and organic molecules). Based on these surface properties and the low *R*_ct_, we suggest that this composite may have theoretical potential for preconcentrating and detecting multiple pollutants. This work demonstrates a way to improve the capacitance of BDD-based electrodes and may serve as a starting point for future exploration in integrated energy-sensing devices after experimental validation.

## 1. Introduction

Integrating energy harvesting, energy storage, and functional sensing onto a single chip or substrate, known as self-powered integrated electrochemical systems, has become an important direction in electrochemistry, microelectronics, and the Internet of Things (IoT) [1,2,3]. Compared with conventional discrete designs, self-powered systems can operate continuously without an external power supply, offering advantages such as miniaturization, light weight, and intelligence [4,5]. They hold promise for applications in wearable health monitoring, real-time environmental sensing, and IoT nodes [6]. In this trend, the integration of energy storage units (e.g., micro-supercapacitors) and sensing units is particularly critical. Storage units require high areal capacitance and low resistance to deliver stable power, while sensing chips need a sensitive interface for rapid and selective detection of trace targets [7,8]. Therefore, developing a versatile electrode material that holds the potential to meet both energy storage and sensing requirements has become a key scientific challenge in this emerging field [9].

Driven by the “one material, two functions” concept, considerable research has been devoted to exploring potential bifunctional electrode materials [10,11,12], including recent studies on laser-induced graphene composites [13], ternary metal oxide heterostructures [14], and vanadium disulfide nanosheets [15]. For example, composites based on laser-induced graphene with silver nanoparticles, ternary metal oxides such as CuO/NiMoO_4_/ZnO, or layered materials like VS_2_ have been explored for combined energy storage and sensing applications [13,14,15]. These studies have demonstrated that judicious material design can enable a single electrode to serve both purposes. However, most of these bifunctional materials rely on metal oxides or carbonaceous substrates, and their stability, potential window, or interfacial charge transfer characteristics may not be optimal for all scenarios [16,17]. Boron-doped diamond (BDD) offers unique advantages—a wide potential window, low background current, and excellent chemical stability—but has rarely been exploited in such bifunctional contexts, especially with a conducting polymer interface engineered for ultra-low charge transfer resistance [18]. Recent studies have also highlighted progress in conductive polymer-based integrated systems [11] and diamond-based supercapacitors [19].

Very recent international investigations have further advanced the field of bifunctional electrodes for integrated energy storage and sensing. For instance, Prakash et al. developed a laser-induced graphene–Ag nanoparticle composite that serves simultaneously as a supercapacitor electrode and an anti-fouling sensor [13]. Miao et al. reported a CuO/NiMoO_4_/ZnO ternary nanocomposite with excellent performance for both dopamine photoelectrochemical sensing and supercapacitors [14]. In the area of conductive polymers, Chen et al. systematically reviewed flexible supercapacitor–sensor integrated systems, emphasizing the critical role of interfacial design [11]. The work by Ji et al. on metallic VS_2_ nanosheets further demonstrated the feasibility of using a single material for multiple electrochemical functions [15]. Moreover, the emerging literature on diamond-based composites for sensing and energy storage has been summarized in recent reviews [18,20]. Notably, the study by Khodadadiyazdi et al. in *Small Science* highlighted the importance of asymmetric C 1s fitting in carbon-based bifunctional materials [21], which has informed our XPS analysis (see Section 3.1). These recent contributions underscore the growing global interest in developing materials that can store energy and detect analytes on the same platform. However, most of these systems still face challenges in achieving simultaneously ultra-low charge transfer resistance and high chemical stability under harsh aqueous conditions.

Based on these advantages, BDD electrodes have been widely studied for both electroanalysis and supercapacitors [20,22]. However, planar BDD electrodes suffer from two critical limitations: low specific surface area and sluggish interfacial charge transfer [23,24]. These limitations restrict both the energy density of BDD-based supercapacitors and the sensitivity of BDD-based electrochemical sensors [25,26]. Hence, improving the surface area and interfacial reaction kinetics of BDD, and developing a BDD-based electrode material that offers both high capacitance and sensing potential, has become an important scientific question in BDD-based bifunctional devices [27].

Two main strategies have been explored to address these limitations. One is to construct three-dimensional porous structures to increase the effective area [28,29]. For example, depositing BDD on porous titanium foam provides more active sites and enhances double-layer capacitance [19]. The other strategy is to modify the BDD surface with pseudocapacitive materials such as metal oxides (e.g., MnO_2_, RuO_2_) or conducting polymers (e.g., polypyrrole (PPy), polyaniline) to introduce additional redox activity [30,31]. Nevertheless, most studies have adopted only one of these strategies [32]. Systematic work combining a high-surface-area porous BDD scaffold with a well-designed surface modification layer to synergistically enhance both capacitance and interfacial charge transfer remains limited [33].

In this work, we prepared a porous BDD film on titanium foam by hot-filament chemical vapor deposition, and then grew PPy layers on the BDD surface by in situ oxidative polymerization. The polymerization time was varied from 8 to 20 h. Furthermore, we provide a theoretical analysis of the feasibility of using this composite for pollutant sensing.

## 2. Materials and Methods

### 2.1. Preparation of Porous BDD and BDD/PPy Films

**Figure 1:**
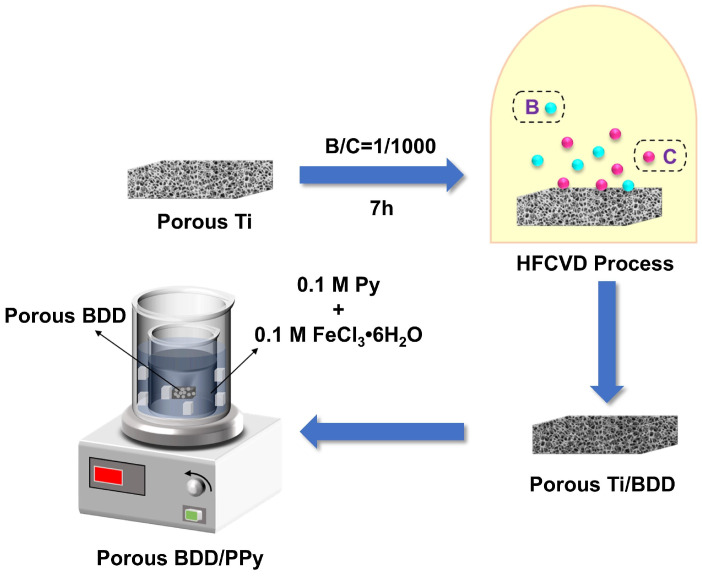
Schematic diagram of the preparation of porous BDD/PPy film.

Figure 1 shows schematic diagram of preparation of porous BDD/PPy films. The porous BDD films were grown on titanium foam (10 × 15 mm^2^, 30 μm pore size) by a system of hot-filament chemical vapor deposition (HFCVD, HF-800, Beijing Worldia, Beijng, China). Ethanol was used as the carbon source (0.26 sccm) with a B/C ratio of 1/1000. Deposition was carried out at 3000 Pa and 700 °C for 7 h.

BDD/PPy composite films were prepared by ice-bath oxidative polymerization. A 0.1 M pyrrole solution and a 0.1 M FeCl_3_·6H_2_O solution were mixed in a 100 mL beaker. The BDD film was immersed in the mixture and stirred in an ice water bath. After the reaction, the sample was removed, rinsed with deionized water, and dried at 40 °C for 0.5 h. The polymerization time was set to 8 h, 12 h, 16 h, and 20 h. The corresponding samples were labeled as BDD/PPy-8 h, BDD/PPy-12 h, BDD/PPy-16 h, and BDD/PPy-20 h. For a detailed step-by-step synthesis protocol, see Section S1 (Supporting Information).

### 2.2. Structural Characterization

X-ray diffraction (XRD, D8-Advance, Bruker AXS, Karlsruhe, Germany) was performed with Cu Kα radiation (λ = 1.5418 Å). The diffraction patterns were recorded over a 2*θ* range of 10–80° with a step size of 0.02° and a scan speed of 2°/min. Surface morphology was observed by scanning electron microscopy (SEM, EVO 18, Carl Zeiss, Jena, Germany) at an acceleration voltage of 15 kV. To enhance conductivity, the samples were sputter-coated with a thin gold layer prior to imaging. Elemental distribution was analyzed using energy dispersive spectroscopy (EDS, Oxford X-Max, Oxford Instruments, Abingdon, UK) attached to the SEM, with data collected at the same accelerating voltage. Raman spectra (LabRAM HR Evolution, Jobin Yvon, Palaiseau, France) with a 532 nm laser as the excitation source. To avoid sample heating or degradation, the laser power was kept below 1 mW. The spectra were collected in the range of 600–2000 cm^−1^ with a grating of 1800 lines/mm.

X-ray photoelectron spectroscopy (XPS, ESCALAB 250Xi, Thermo Fisher Scientific, Waltham, MA, USA) was carried out using monochromatic Al Kα radiation (1486.6 eV). Survey spectra were recorded with a pass energy of 100 eV and a step size of 0.5 eV, while high-resolution spectra (C 1s, N 1s) were measured with a pass energy of 20 eV and a step size of 0.1 eV. Binding energies were calibrated using the adventitious C 1s peak at 284.8 eV. A dual-beam charge neutralization system (low-energy electron flood gun + low-energy Ar^+^ ions) was applied to avoid surface charging. Data analysis and peak fitting were performed using CasaXPS software (version 2.3.25) with a Shirley background and Gaussian–Lorentzian mixed functions (30% Lorentzian). The excitation energy was monochromatic Al Kα (1486.6 eV). For survey spectra, the pass energy was 100 eV with a step size of 0.5 eV (energy resolution ≈ 1.0 eV); for high-resolution C 1s and N 1s spectra, the pass energy was 20 eV with a step size of 0.1 eV (resolution ≈ 0.5 eV). All binding energies were calibrated using the adventitious C 1s peak at 284.8 eV; the calibration was independently verified using the Au 4f_7/2_ peak (84.0 eV) from a gold reference sample. To avoid surface charging during XPS acquisition, a dual-beam charge neutralization system (low-energy electron flood gun combined with low-energy Ar^+^ ion beam) was applied. The effectiveness of charge neutralization was confirmed by the stable position of the C 1s peak at 284.8 eV (variation within ±0.1 eV) and the absence of peak broadening or distortion. Peaks were fitted after Shirley background subtraction. Initial peak positions were constrained based on literature values, and the full widths at half maximum (FWHM) were allowed to vary within ±0.2 eV of the initial guesses. Fitting converged when the chi-square value reached a minimum, and the residual errors for all peaks were below 3%, confirming good fit quality.

### 2.3. Electrochemical Measurements

Electrochemical tests were carried out using an electrochemical workstation (CHI 660E, CH Instruments, Shanghai, China) in a three-electrode configuration, where the porous BDD/PPy film served as the working electrode, a platinum sheet (10 × 15 mm^2^) as the counter electrode, and an Ag/AgCl (3 M KCl) as the reference electrode. Cyclic voltammetry (CV), galvanostatic charge–discharge (GCD), and electrochemical impedance spectroscopy (EIS) were carried out to evaluate the electrochemical performance. All electrochemical measurements were performed on three independently prepared electrodes, and the data are expressed as the mean ± standard deviation (SD). Specifically, for each polymerization time (8, 12, 16, 20 h), three independent synthesis batches were carried out. From each batch, one electrode was fabricated and measured in triplicate. The reported values are the mean ± SD calculated from the three batch means, representing batch-to-batch reproducibility.

## 3. Results and Discussion

### 3.1. Morphological and Structural Characterization

As shown in the XRD patterns (Figure S1a,b), the diamond structure of BDD remains unchanged after PPy deposition, and PPy is amorphous. Figure S1c,d show the morphology of the Ti foam, the porous BDD film, and the BDD/PPy composite. The Ti foam has a three-dimensional macroporous skeleton with a pore size of about 30 μm (Figure S1c). After HFCVD, BDD grains (B/C = 1/1000) uniformly cover the Ti skeleton, forming a porous BDD layer (Figure S1d). The diamond grains are angular, with an average size of about 0.69 μm. This rough, porous, and conductive BDD layer provides a good substrate for subsequent PPy deposition.

**Figure 2:**
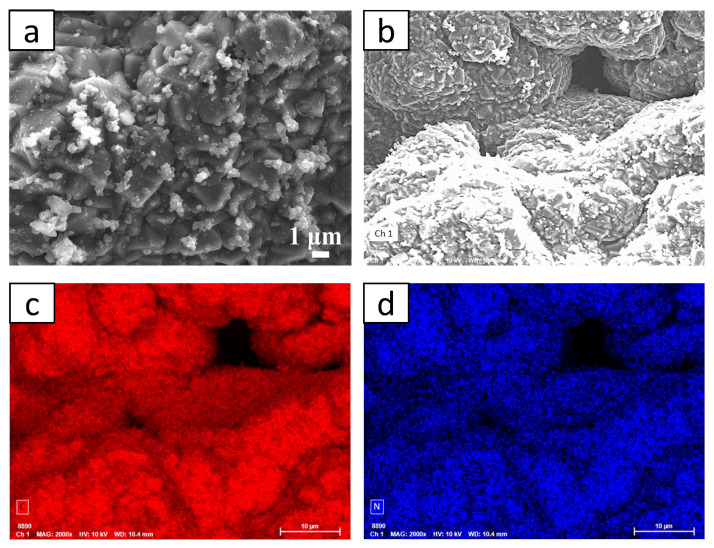
BDD/PPy-12 h film’s (**a**) SEM image. (**b**–**d**) EDS mapping showing C and N distributions (the microscope’s magnification or scale bar are consistent).

**Figure 3:**
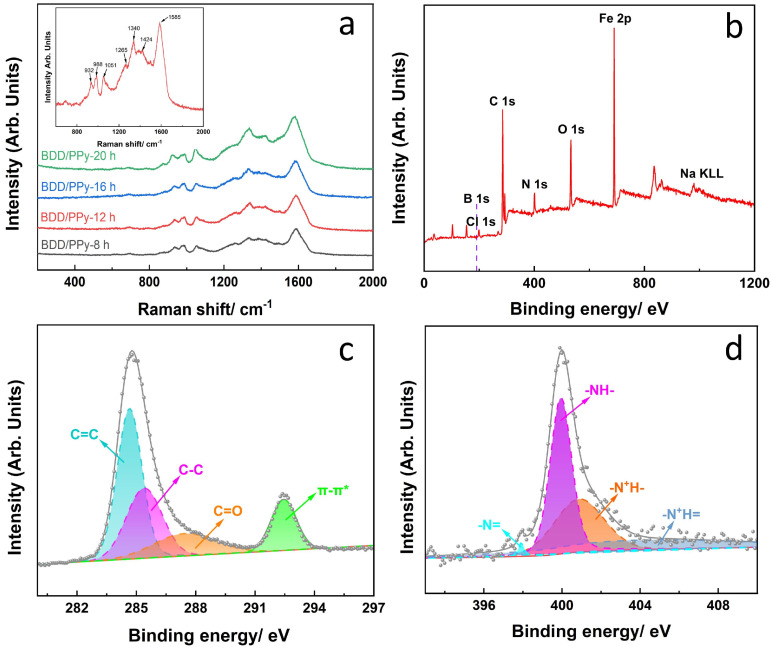
(**a**) Raman spectra of BDD/PPy films under four polymerization times; Insert: expanded view of the 600–2000 cm^−1^ region. (**b**) Full survey XPS spectrum (0–1200 eV) of the BDD/PPy-12 h thin film. (**c**) High-resolution C 1s spectrum. (**d**) High-resolution N1s spectrum.

Figure 2a shows the BDD/PPy composite obtained after 12 h of polymerization. The particles are well distributed without significant agglomeration, leaving gaps that may help ion transport. Quantitative particle size analysis (Figure S2a) confirms that the 12 h sample has a uniform average diameter of ~260 nm, while longer polymerization leads to slight growth and broader distribution (Figure S2). This structure creates a hierarchical pore system consisting of micrometer-sized pores from the Ti/BDD framework and nanoscale gaps between PPy spheres. EDS mapping (Figure 2b–d) shows that C and N (the latter characteristic of PPy) are distributed in the same regions, confirming that PPy uniformly coats the BDD surface. The elemental composition is supported by both the XPS results (Table S1) and the element distribution maps (Figure S3). Raman spectra (Figure 3a) show peaks at about 1585 cm^−1^ (C=C backbone) and 1340 cm^−1^ (C–N stretching) from PPy [34,35,36]. The insert in Figure 3a indicates that the diamond *sp*^3^-C peak at about 1332 cm^−1^ is weak, confirming the presence of PPy on the BDD surface.

**Figure 4:**
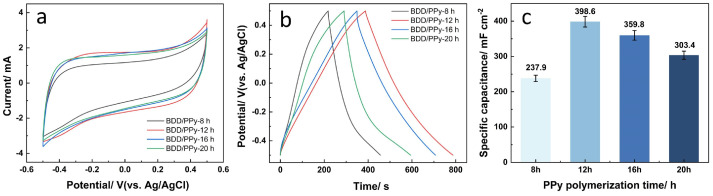
Electrochemical performance of BDD/PPy electrode under four polymerization times. (**a**) Four CV curves at a scan rate of 10 mV/s. (**b**) Four GCD curves at a current density of 1 mA/cm^2^. (**c**) The capacitance calculated by GCD curve. Data are mean ± SD (*n* = 3 independent batches).

The full survey XPS spectrum (Figure 3b, 0–1200 eV) shows intense C 1s, N 1s, and O 1s peaks; the N element originates from PPy. The residual plots for C 1s and N 1s fits (Figure S4a,b) show randomly distributed residuals around zero, confirming the high quality of the peak fitting. In addition, weak Fe 2p (~710 eV) and Cl 2p (~200 eV) peaks are visible. Quantitative XPS analysis (Table S1) reveals residual Fe and Cl (<0.5 at%) originating from the FeCl_3_ oxidant. A small Na KLL peak (~980 eV) is also present, likely from trace electrolyte residues. No additional redox peaks are observed in the CV curves (Figure 4a), suggesting that these residues do not appreciably affect the electrochemical performance under the present conditions. In the 100–200 eV region, very weak Si 2p (~103 eV) and Si 2s (~154 eV) peaks are observed, which originate from trace glassware contamination during sample preparation. These impurity signals are negligible and do not affect the quantitative analysis of the main elements (C, N, O). The high-resolution C 1s spectrum (Figure 3c) is fitted to four components: C=C (284.6 eV), C–C (285.3 eV), C=O (287.5 eV), and π-π* (292.4 eV) [37,38,39]. The N 1s spectrum (Figure 3d) shows four peaks: pyrrolic –NH– at 399.9 eV, polaronic –N^+^H– at 401.0 eV, bipolaronic =N^+^H– at 404.2 eV, and iminic –N= at 397.8 eV [34,35,40]. The presence of protonated nitrogen species (–N^+^H– and =N^+^H–) is consistent with the conductive form of PPy. The pyrrolic –NH– groups are known to chelate metal ions and some organic pollutants, which may be useful for preconcentration in sensing applications. The quantitative atomic percentages derived from XPS are summarized in Table S1.

### 3.2. Electrochemical Performance

We first examined how the PPy polymerization time affects the electrochemical behavior. Figure 4a shows CV curves at 10 mV/s for BDD/PPy electrodes prepared with 8, 12, 16, and 20 h. All curves have a quasi-rectangular shape without distinct redox peaks, indicating good capacitive behavior and reversibility. The BDD/PPy-12 h electrode gives the largest enclosed area, suggesting the highest specific capacitance.

(1) $$C_{s}=\frac{I\times ∆t}{S\times ∆V}$$

where *I* is the applied current (A), Δ*t* is the discharge time (s), *S* is the effective wetted area of the working electrode (cm^2^), and Δ*V* is the potential window (V).

Figure 4b presents GCD curves at 1 mA/cm^2^. The symmetrical triangular shapes indicate good reversibility. From Equation (1), the specific capacitances are 237.9 ± 9.1, 398.6 ± 15.2, 359.8 ± 13.7, and 303.4 ± 11.6 mF/cm^2^ (Figure 4c). The 12 h sample gives the highest value, which is 5.8 times that of the porous BDD electrode without PPy (67.9 mF/cm^2^). Although the HFCVD process is time-consuming, the electrochemical parameters herein (including capacitance, *R*_s_, and *R*_ct_) were evaluated based on three independently prepared batches (*n* = 3), following the same strictly controlled fabrication conditions. The significant performance advantage at 12 h was reproducible across all batches and clearly exceeds routine experimental variations. The improvement at 12 h is attributed to a uniform and conformal PPy coating that increases the electroactive area and provides pseudocapacitance without blocking ion pathways. At 8 h, the PPy loading is insufficient; at 16 and 20 h, excess PPy leads to agglomeration, hindering electrolyte infiltration [41].

**Figure 5:**
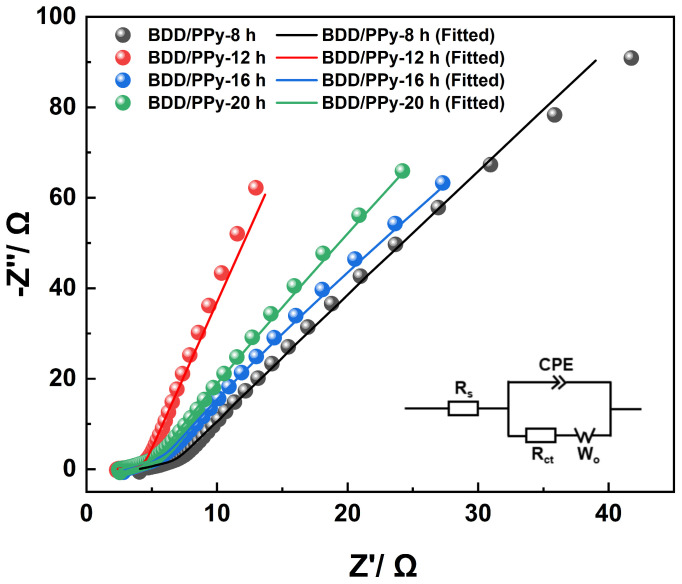
Impedance spectra of BDD/PPy thin film electrodes under four polymerization times.

EIS spectra (Figure 5) were recorded from 0.01 Hz to 100 kHz with an AC amplitude of 5 mV at the open-circuit potential. All spectra consist of a depressed semicircle in the high-frequency region followed by a linear tail in the low-frequency region, indicating a combination of charge-transfer and diffusion-controlled processes. The experimental data were fitted using the equivalent circuit *R*_s_(CPE(*R*_ct_W_o_)) (inset of Figure 5). In this circuit, *R*_s_ represents the series resistance (electrolyte and contact resistance), CPE is a constant phase element accounting for the non-ideal porous electrode capacitance, *R*_ct_ is the charge transfer resistance, and W_o_ is the Warburg impedance describing semi-infinite diffusion. The fitted curves are overlaid as solid lines in Figure 5; an excellent agreement is observed for all samples, with chi-square (χ^2^) values below 1 × 10^−3^ (see Table S2 for detailed parameters and their errors).

For BDD/PPy-12 h, *R*_s_ = 2.3 ± 0.2 Ω and *R*_ct_ = 1.3 ± 0.1 Ω, the lowest among the four samples. To further evaluate the capacitive behavior, Bode plots were constructed from the same EIS data and are presented in Figure S5. The BDD/PPy-12 h electrode shows a phase angle of approximately −78° at 0.01 Hz (Figure S5a), which is very close to the ideal −90° for a pure capacitor, confirming its low diffusion resistance and superior capacitive performance. This result is consistent with the steepest low-frequency line in the Nyquist plot for the 12 h sample (approximately 70° estimated from the slope). In contrast, the other electrodes exhibit lower phase angles at low frequencies, indicating higher diffusion impedance. The Bode modulus plots (Figure S5b) show that |Z| decreases with increasing frequency, and the 12 h sample has the lowest |Z| in the low-frequency region, further supporting its fast ion diffusion and charge transfer kinetics. The low *R*_ct_ is attributed to the dual electron conduction pathways through the BDD substrate and the PPy layer.

**Figure 6:**
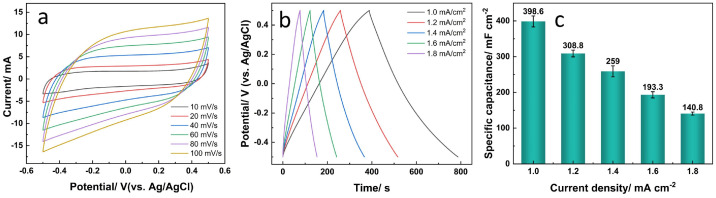
Electrochemical performance of BDD/PPy-12 h thin film electrode (**a**) CV curve of 10–100 mV/s. (**b**) GCD curves of 1.0–1.8 mA/cm^2^. (**c**) The variation graph of specific capacitance calculated by GCD. Data are mean ± SD (*n* = 3 independent batches).

Figure 6a shows CV curves of the BDD/PPy-12 h electrode at scan rates from 10 to 100 mV/s. The curves maintain a quasi-rectangular shape with good symmetry. Specific capacitance decreases from 353.2 mF/cm^2^ at 10 mV/s to 187.8 mF/cm^2^ at 100 mV/s, and the capacity retention rate is 53%. Figure 6b shows GCD curves at current densities from 1 to 1.8 mA/cm^2^; the triangular shapes remain symmetrical. Corresponding capacitances are shown in Figure 6c. The decrease at higher rates is typical and results from limited time for ions to diffuse into the inner active sites [42].

Table 1 compares the performance of BDD/PPy-12 h with other BDD-based and PPy-based electrodes. The areal capacitance (398.6 ± 15.2 mF/cm^2^) exceeds that of most pristine BDD electrodes and is comparable to some composites. More importantly, the *R*_ct_ of 1.3 ± 0.1 Ω is substantially lower than that of porous BDD (81 Ω), Ti/BDD (4.8 Ω), BDD-NSs-5 (62.1 Ω), pure PPy (142.3 Ω), and PPy/MXene (49.5 Ω). This low interfacial resistance is a key feature of the present work.

**Figure 7:**
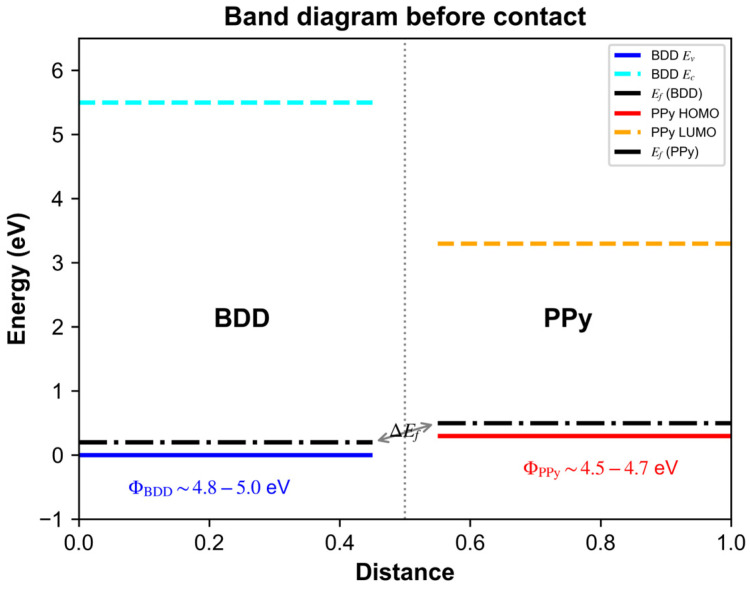
Energy band diagram of the BDD/PPy interface before contact. *E*_v_: valence band maximum, *E*_c_: conduction band minimum, *E*_f_: Fermi level, HOMO: highest occupied molecular orbital, LUMO: lowest unoccupied molecular orbital. The work function alignment hypothetically leads to a low charge transfer barrier. This band diagram is a schematic illustration based on literature values; direct experimental verification of the interface properties is beyond the scope of this work.

To hypothetically understand the origin of the ultra-low *R*_ct_ in the BDD/PPy-12 h composite, we analyze the electronic band structures of BDD and PPy. As shown in Figure 7, p-type BDD has a work function of about 4.8–5.0 eV (Fermi level near valence band), while PPy has a work function of about 4.5–4.7 eV. Upon contact, the Fermi levels align, creating a narrow depletion region that facilitates carrier tunneling and minimizes interfacial resistance. Figure S6 further benchmarks our composite against representative electrodes from the literature. Notably, the *R*_ct_ of 1.3 Ω even outperforms some high-performance composites such as porous BDD/MnO_2_ (2 Ω) and BG/BDD (2.4 Ω). This combined analysis suggests that the synergistic interfacial engineering could provide fast electron pathways and might ensure a favorable energy level alignment, potentially contributing to the exceptionally low *R*_ct_. However, direct experimental verification of these mechanisms (e.g., work function measurements, interface characterization) is beyond the scope of this work.

### 3.3. Future Perspectives: Theoretical Feasibility for Versatile Water Pollutant Sensing

No direct sensing experiments are presented; the following discussion is solely based on material properties (low *R*_ct_ and pyrrolic –NH– groups). The previous sections show that the BDD/PPy-12 h composite has two characteristics that are relevant to electrochemical sensing. First, its charge transfer resistance is very low (*R*_ct_ = 1.3 ± 0.1 Ω). In stripping voltammetry—a common technique for trace pollutant detection—the sensitivity strongly depends on the rate of electron transfer between the electrode and the target species. A lower *R*_ct_ typically allows higher stripping peak currents and faster response. Second, XPS analysis (Figure 3d) reveals the presence of pyrrolic –NH– groups on the PPy surface. These groups have lone pair electrons that can chelate various pollutants, including heavy metal ions (e.g., Pb^2+^, Cd^2+^, Hg^2+^) and some organic molecules with electrophilic sites [45]. This chelation can help preconcentrate pollutants from the bulk solution onto the electrode surface, which is a key step for lowering the detection limit.

Based on these two features—low *R*_ct_ and –NH– chelation—we hypothesize that the BDD/PPy-12 h composite holds theoretical promise as an electrode material for detecting multiple water pollutants. The porous structure may further facilitate mass transport. However, it is crucial to note that rigorous experimental validation (e.g., square-wave anodic stripping voltammetry for metal ions, or amperometric detection for organic pollutants) is needed to confirm this potential, and such work is planned as part of our ongoing research on integrated water monitoring platforms. A brief description of how this material could theoretically be integrated into our “Smart Water Sensing” multi-chip continuous detection device is given in Section S3.

## 4. Conclusions

In summary, we successfully prepared a hierarchical porous BDD/PPy composite electrode by combining hot-filament chemical vapor deposition of BDD on titanium foam with in situ oxidative polymerization of PPy. The polymerization time was systematically varied, and 12 h was identified as the optimal duration, yielding a uniform PPy nanosphere coating (~260 nm diameter) that fully covers the BDD skeleton without excessive agglomeration. This hierarchical architecture provides rapid ion diffusion pathways through the micrometer-sized pores of the Ti/BDD framework and additional pseudocapacitance from the PPy layer, while preserving the structural integrity and conductivity of the BDD network.

The BDD/PPy-12 h electrode achieved an areal capacitance of 398.6 ± 15.2 mF/cm^2^ at 1 mA/cm^2^. Its charge-transfer resistance (*R*_ct_) was as low as 1.3 ± 0.1 Ω. The low *R*_ct_ may be tentatively attributed to the dual electron conduction pathways (through the BDD substrate and the PPy layer) and the favorable energy-level alignment at the BDD/PPy interface, which is suggested by the band structure analysis based on literature values.

X-ray photoelectron spectroscopy confirmed the presence of pyrrolic –NH– groups on the PPy surface, which are known to chelate various water pollutants. Combined with the ultra-low *R*_ct_, these properties suggest theoretical potential of the composite for pollutant preconcentration and detection. However, no direct sensing experiments were performed in this study; experimental validation remains a subject of future work.

Overall, this work demonstrates a viable strategy to overcome the low specific capacitance and sluggish charge transfer of planar BDD electrodes by constructing a porous BDD/PPy heterostructure. The resulting material offers a promising platform for integrated energy storage devices, with potential extensions to sensing applications after further experimental validation.

## Figures and Tables

**Table 1:** Comparison of performances of different electrode materials.

Electrode Material	Specific Capacitance	Current Density	*R*_ct_	Ref.
Porous BDD/PPy	398.6 ± 15.2 mF/cm^2^	1.0 mA/cm^2^	1.3 ± 0.1 Ω	This work
Ti/PPy	45.7 mF/cm^2^	1.2 mA/cm^2^	57.6 Ω	This work
Porous BDD	67.9 mF/cm^2^	0.22 mA/cm^2^	81 Ω	This work
Porous BDD/MnO_2_	1383.6 mF/cm^2^	2 mA/cm^2^	2 Ω	[32]
BDD-NSs-5	47.1 mF/cm^2^	2 mA/cm^2^	62.1 Ω	[19]
Ti/BDD	45.7 mF/cm^2^	0.02 mA/cm^2^	4.8 Ω	[22]
BG/BDD	203.9 F/cm^3^	0.65 A/cm^3^	2.4 Ω	[43]
PPy	0.54 F/cm^2^	2.5 mA/cm^2^	142.3 Ω	[44]
PPy/MXene	1.975 F/cm^2^	2.5 mA/cm^2^	49.5 Ω	[44]

## Data Availability

The data presented in this study are available on request from the corresponding author.

## Supplementary Materials

The following supporting information can be downloaded at: https://www.mdpi.com/article/10.3390/nano16120755/s1, S1: Detailed Synthesis Protocol; S2: Additional Data on Morphological and Structural Characterization; Figure S1: XRD spectra of (a) BDD thin films at four boron doping concentrations and (b) BDD/PPy films under four polymerization times. SEM image of (c) Ti_30_/BDD film and (d) BDD/PPy-B/C = 1/1000; Figure S2: (a) Particle size distribution histogram of BDD/PPy-12 h based on measurements from three random regions. SEM images of BDD/PPy films under three polymerization times: (b) BDD/PPy-8 h; (c) BDD/PPy-16 h; (d) BDD/PPy-20 h; Figure S3: EDS element mapping of the BDD/PPy-12 h composite film. (a) Overlay of C (red), O (green), Ti (pink), and N (blue); (b) oxygen (O) distribution; (c) titanium (Ti) distribution; Figure S4: Residual plots for the (a) C 1s and (b) N 1s XPS spectra of the BDD/PPy-12 h composite. The residuals are randomly distributed around zero, and the relative fitting error is below 3%, confirming the high quality of the fits; Table S1: XPS quantitative analysis of the BDD/PPy-12 h composite; S3: Additional Data on Electrode Performance; Figure S5: Bode plots of the BDD/PPy-12 h electrode: (a) phase angle versus frequency; (b) impedance modulus versus frequency. The phase angle at 0.01 Hz is −78°, and the |Z| value decreases as frequency increases, confirming good capacitive behavior. Measurement conditions: 0.1 M Na_2_SO_4_, OCP, AC amplitude 5 mV; Table S2: Equivalent circuit fitting parameters for BDD/PPy electrodes; Figure S6: Comparison of charge transfer resistance (*R*_ct_) of BDD/PPy-12h with other BDD-based and PPy-based electrodes. Data from Table 1 and references. The data in red represents this work, showing the lowest *R*_ct_ value (1.3 Ω); S4: Potential Integration into “Smart Water Sensing” Multi-Chip Continuous Detection Platform.
