# Supplementary material for: BDD/PPy Composites with Low Interfacial Resistance for Energy Storage and Theoretical Feasibility for Pollutant Sensing
Source: Nanomaterials (Basel). 2026 Jun 16;16(12):755. doi: 10.3390/nano16120755 (PMC13304678; doi:10.3390/nano16120755)
Supplement: Supplementary file 1 [file nanomaterials-16-00755-s001.zip › nanomaterials-4335661-supplementary.pdf]

Supplementary Materials

BDD/PPy Composites with Low Interfacial Resistance for Energy Storage and Theoretical Feasibility for Pollutant Sensing

Shuhan Wang[1,†], Yifan Ren[1,†], Qinghai Yu[2,*], Jiarui Yang[1], Jiali Lin[1], Lingpei Shi[1] and Yuanyuan Li[1].

[1] School of Materials Science and Technology, China University of Geosciences (Beijing), Beijing 100083, China; shuhan_wang@email.cugb.edu.cn (S.W.); yifan_ren@email.cugb.edu.cn (Y.R.); jiarui_yang@email.cugb.edu.cn (J.Y.); jiali_lin@email.cugb.edu.cn (J.L.); lingpeishi@email.cugb.edu.cn (L.S.); 2464770458@qq.com (Y.L.)

[2] School of Gemmology, China University of Geosciences (Beijing), Beijing 100083, China

* Correspondence: yqh@cugb.edu.cn

† These authors contributed equally to this work.

S1. Detailed Synthesis Protocol

Ti foam pretreatment: The Ti foam (10 × 15 mm^2, 30 μm pore size) was ultrasonically cleaned in acetone (10 min), ethanol (10 min), and deionized water (10 min), then dried under N_2 flow.

Diamond seeding: The cleaned foam was immersed in a nanodiamond suspension (5 g/L, grain size ~5 nm) for 30 min, followed by ultrasonic agitation for 15 min to achieve uniform coverage.

HFCVD parameters: Ethanol flow rate: 0.26 sccm; H_2 flow rate: 200 sccm; trimethyl borate flow rate: 0.0026 sccm (to achieve B/C=1/1000). Filament-substrate distance: 8 mm. Deposition time: 7 h. BDD thickness was estimated from cross-section SEM as ~2 μm.

PPy polymerization: The pyrrole/$FeCl_3$ mixture (20 mL each) was stirred at 400 rpm. After polymerization, the sample was washed with deionized water (3 × 20 mL). PPy loading (mass gain) was measured using a microbalance (0.01 mg accuracy). PPy thickness was estimated from SEM cross-section images as ~260 nm for the 12 h sample (the value reported in the main text). Residual Fe and Cl were detected by XPS (see main text Figure 3b and Table S2) and found to be < 1 at%.

S2. Additional Data on Morphological and Structural Characterization

Figure S1a and S1b show the morphology of the Ti foam and the porous BDD film. XRD patterns (Figures S1c and S1d) show diamond peaks at 43.9° (111) and 75.3° (220), indicating that the BDD crystal structure remains unchanged after PPy deposition. Peaks at 36.1°, 41.8°, 60.8°, and 72.7° correspond to TiC formed at the Ti–BDD interface, which helps adhesion. No distinct PPy diffraction peaks are observed, suggesting that PPy is amorphous.

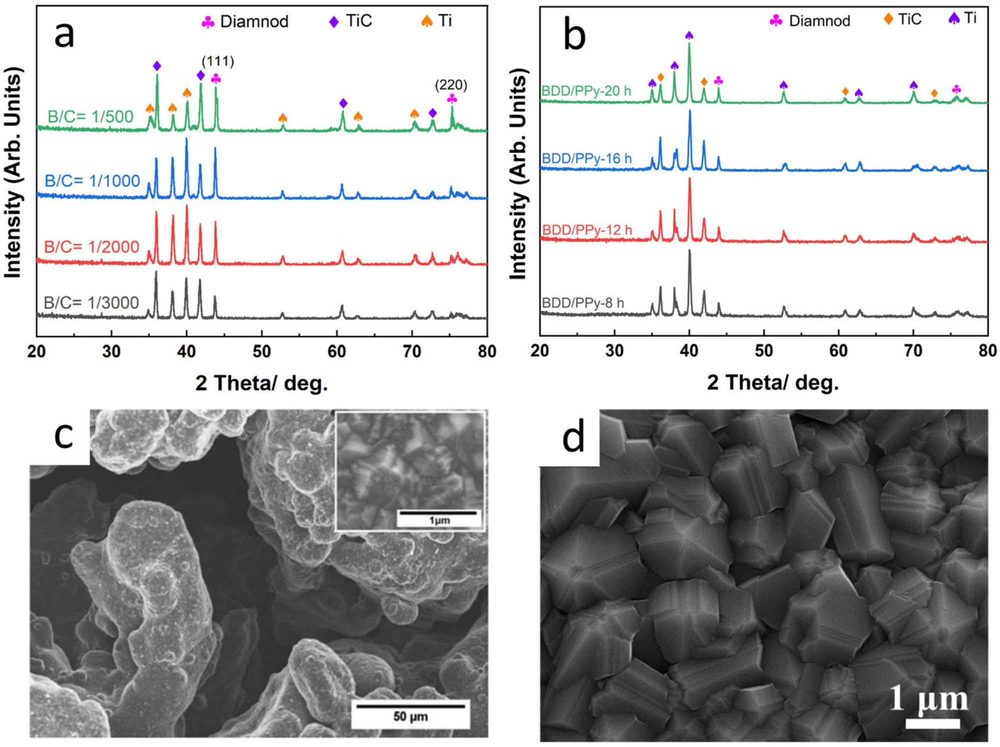

Figure S1. XRD spectra of (**a**) BDD thin films at four boron doping concentrations and (**b**) BDD/PPy films under four polymerization times. SEM image of (**c**) Ti_{30}/BDD film and (**d**) BDD/PPy-B/C = 1/1000.

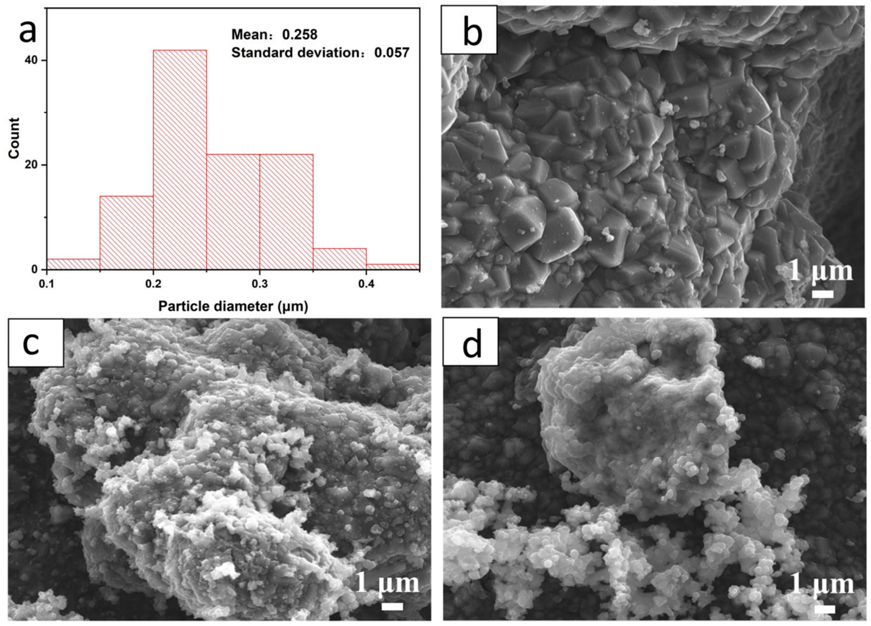

Figure S2. (**a**) Particle size distribution histogram of BDD/PPy-12 h based on measurements from three random regions. SEM images of BDD/PPy films under three polymerization times: (**b**) BDD/PPy-8 h; (**c**) BDD/PPy-16 h; (**d**) BDD/PPy-20 h.

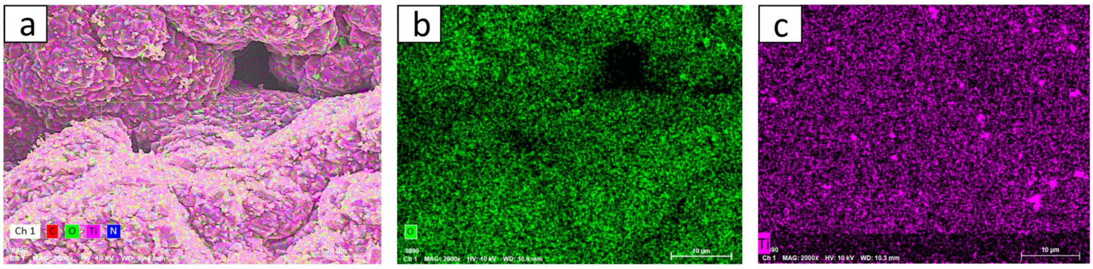

Figure S3. EDS element mapping of the BDD/PPy-12 h composite film. (**a**) Overlay of C (red), O (green), Ti (pink), and N (blue); (**b**) oxygen (O) distribution; (**c**) titanium (Ti) distribution.

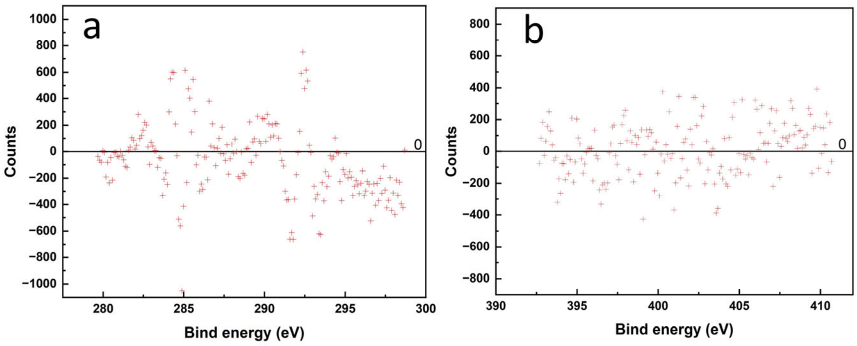

Figure S4. Residual plots for the (**a**) C 1s and (**b**) N 1s XPS spectra of the BDD/PPy-12 h composite. The residuals are randomly distributed around zero, and the relative fitting error is below 3%, confirming the high quality of the fits.

Table S1. XPS quantitative analysis of the BDD/PPy-12 h composite.

Element/Component	BE (eV)	Assignment	Atomic% (relative)
C 1s (total = 63.13%)			
C1	284.6	C=C (sp^2)	42.46
C2	285.3	C–C (sp^3)	27.89
C3	287.5	C=O	15.33
C4	292.4	π–π* shake-up satellite	14.33
N 1s (total = 8.29%)			
N1	397.8	Iminic –N=	2.30
N2	399.9	Pyrrolic –NH–	48.50
N3	401.0	Polaronic –N^+H–	36.00
N4	404.2	Bipolaronic =N^+H–	13.20
O 1s	~532	C–O, C=O	14.89

B 1s	~187	B–C	<0.5
Fe 2p	~710	residual Fe	<0.5
Cl 2p	~200	residual Cl	<0.5

*Note: The B 1s signal is weak and its quantification is subject to large uncertainty due to low sensitivity and possible surface contamination. The nominal B/C ratio in the precursor was 1/1000, consistent with the very low B content observed in XPS. The trace Fe and Cl residues originate from the $FeCl_3$ oxidant. Their potential influence on electrochemical performance (e.g., pseudocapacitance, conductivity, or charge transfer resistance) has not been experimentally isolated; however, their very low concentrations (<0.5 at% each) suggest any such effect is likely minor.

S3. Additional Data on Electrode Performance

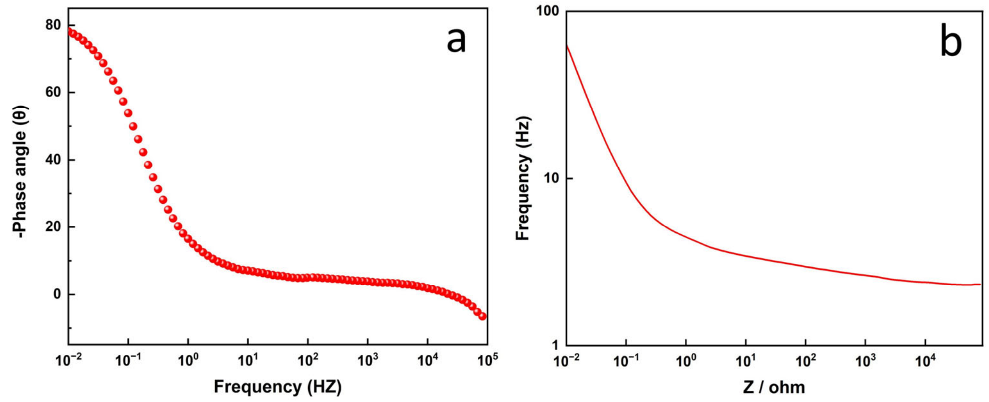

Figure S5. Bode plots of the BDD/PPy-12 h electrode: (a) phase angle versus frequency; (b) impedance modulus versus frequency. The phase angle at 0.01 Hz is −78°, and the |Z| value decreases as frequency increases, confirming good capacitive behavior. Measurement conditions: 0.1 M Na_2SO_4, OCP, AC amplitude 5 mV.

The pristine porous BDD electrode (without PPy) was tested under the same conditions. Its areal capacitance was 67.9 mF/cm^2 at 0.22 mA/cm^2, which serves as the baseline for comparison. The effect of PPy loading was also examined by SEM. At a polymerization time of 8 h, the PPy coating was sparse and did not fully cover the BDD surface. At 16 h and 20 h, PPy particles tended to agglomerate, potentially blocking ion transport pathways. These morphological Table S2 summarizes the series resistance (R_s) and charge transfer resistance (R_{ct}) obtained from fitting the EIS spectra in Figure 5.

Table S2. Equivalent circuit fitting parameters for BDD/PPy electrodes.

Sample	R_s (Ω)	R_{ct} (Ω)	CPE ($Ω^{-1}$ s^n)	n	$χ^2$
BDD/PPy-8 h	4.1 ± 0.3	1.9 ± 0.2	1.5×10^{-3}	0.85	4.2×10^{-4}
BDD/PPy-12 h	2.3 ± 0.2	1.3 ± 0.1	1.2×10^{-3}	0.89	4.8×10^{-4}
BDD/PPy-16 h	2.8 ± 0.2	1.7 ± 0.2	1.3×10^{-3}	0.87	5.1×10^{-4}
BDD/PPy-20 h	2.6 ± 0.2	1.5 ± 0.2	1.4×10^{-3}	0.86	4.9×10^{-4}

Data are mean ± SD (n=3 independent batches).

To visually compare the R_{ct} values of our material with other reported materials, a bar chart is presented in Figure S2. The corresponding numerical values are given in Table 1 in the main text. As clearly shown, our material outperforms all others, exhibiting the smallest R_{ct}.

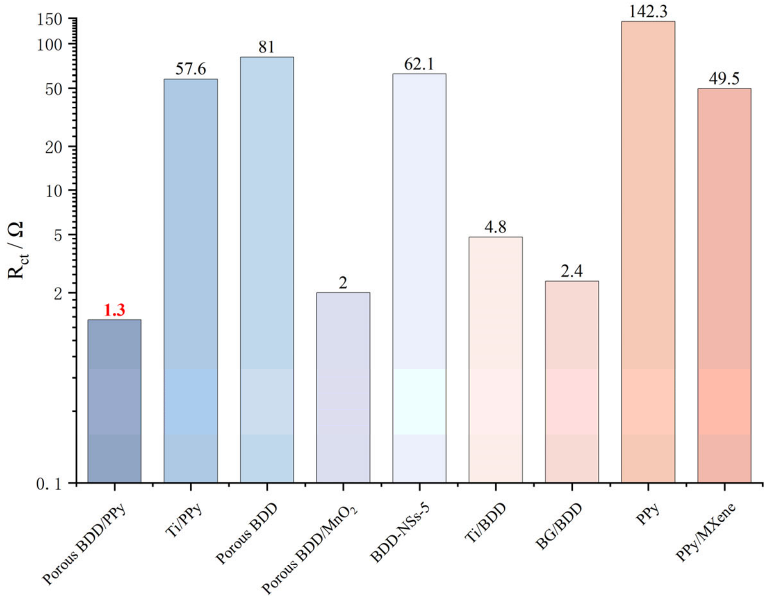

Figure S6. Comparison of charge transfer resistance (R_{ct}) of BDD/PPy-12h with other BDD-based and PPy-based electrodes. Data from Table 1 and references. The data in red represents this work, showing the lowest R_{ct} value (1.3 Ω)

S4. Potential Integration into "Smart Water Sensing" Multi-Chip Continuous Detection Platform

Our research group ("Smart Water Sensing" team) has been developing a water quality monitoring system that combines a multifunctional SERS substrate, microfluidics, and a rotary multi-chip continuous detection device. The system uses an AI-based visualization platform and a "3T+1P" intelligent sensing strategy to detect multiple pollutants (heavy metals, organic compounds, etc.) with high sensitivity and throughput in real time.

The core products include: (i) An in-situ water quality monitor (patented); (ii) A smart monitoring wristband (patent pending); and (iii) A sewage online detection simulation software (V1.0). These three components form a "fixed + portable + virtual" three-dimensional monitoring system.

The following discussion is a forward-looking perspective and does not constitute experimental demonstration. The BDD/PPy-12 h composite electrode developed in this work has a low charge transfer resistance (1.3 ± 0.1 Ω) and pyrrolic –NH– groups that is expected to chelate various pollutants. These properties make it a promising candidate for the sensing element in the above devices. For example, the electrode could be integrated into the rotary multi-chip module for sequential detection of different pollutants. Experimental validation of the sensing performance (e.g., sensitivity, detection limit, selectivity) is ongoing and will be reported in future work.
